# Recent advances in N-glycan biomarker discovery among human diseases: N-glycan biomarkers in diseases

**DOI:** 10.3724/abbs.2024101

**Published:** 2024-06-21

**Authors:** Yi Wang, Yuanyuan Liu, Si Liu, Liming Cheng, Xin Liu

**Affiliations:** 1 Department of Laboratory Medicine Tongji Hospital Tongji Medical College Huazhong University of Science and Technology Wuhan 430030 China; 2 The Key Laboratory for Biomedical Photonics of MOE at Wuhan National Laboratory for Optoelectronics-Hubei Bioinformatics & Molecular Imaging Key Laboratory Systems Biology Theme Department of Biomedical Engineering College of Life Science and Technology Huazhong University of Science and Technology Wuhan 430074 China; 3 Department of Epidemiology and Health Statistics School of Public Health Fujian Medical University Fuzhou 350122 China

**Keywords:** N-glycan, glycomics, biomarker, diagnosis, prognosis

## Abstract

N-glycans play important roles in a variety of biological processes. In recent years, analytical technologies with high resolution and sensitivity have advanced exponentially, enabling analysts to investigate N-glycomic changes in different states. Specific glycan and glycosylation signatures have been identified in multiple diseases, including cancer, autoimmune diseases, nervous system disorders, and metabolic and cardiovascular diseases. These glycans demonstrate comparable or superior indicating capability in disease diagnosis and prognosis over routine biomarkers. Moreover, synchronous glycan alterations concurrent with disease initiation and progression provide novel insights into pathogenetic mechanisms and potential treatment targets. This review elucidates the biological significance of N-glycans, compares the existing glycomic technologies, and delineates the clinical performance of N-glycans across a range of diseases.

## Introduction

In recent years, the importance of glycobiology in human diseases has been increasingly emphasized due to its numerous biological functions in animal systems. Glycosylation, the most abundant and complex form of posttranslational modification, involves the addition of complex oligosaccharide structures to a broad spectrum of molecules, including proteins and lipids [1]. Glycans can attach to polypeptide structures via amide linkages to Asn side chains (referred to as N-glycosylation), glycosidic linkages to Ser/Thr, Lys, or Tyr side chains (referred to as O-glycosylation), or C-C linkages to the C2 position of Trp (referred to as C-mannosylation) [1]. This review will focus on N-glycans, the most extensively studied glycosylated forms in eukaryotic organisms.

By modifying the characteristics of the attached molecules, N-glycosylation facilitates the modulation of diverse biological events. Typically, glycan structures on functional proteins contribute to regulating cytoplasmic and nuclear functions, immune surveillance, inflammatory responses, and hormonal effects [2]. Furthermore, glycosylations on cell surface proteins can directly regulate their functions, thereby impacting both intracellular signal transduction and interactions with the extracellular environment [ 2‒ 4]. In pathological processes, specific alterations in glycan synthesis and glycosylation pathways result in the production of abnormal glycoproteins, which not only offer partial explanations for pathogenic mechanisms but can also indicate the disease state [ 5, 6]. To date, genome-wide association studies (GWASs) have highlighted the pathogenic effects of glycosylation on several complex disorders, including autoimmune diseases, inflammatory diseases, and type 1 diabetes [ 7‒ 9]. In addition, aberrant N-glycans have been observed in a range of diseases, while in healthy individuals, the abundance of N-glycans remains surprisingly stable, with exceptions for changes due to aging, diseases, or lifestyle adjustments [ 10‒ 12]. Remarkably, the glycosylation changes related to disease often precede other symptoms, highlighting their significant potential as early indicators of disease. However, only a few N-glycans have been approved for clinical application in the context of cancer diagnosis and therapy, and most of them are still in research or clinical translation.

In this review, we have summarized the recent advances in N-glycan biomarkers for various diseases, with the aim of clarifying the current status of research and identifying challenges to be addressed to provide guidance for future investigations and facilitate clinical translation.

## Human Glycosylation

### Biosynthesis of glycans

**Figure 1:**
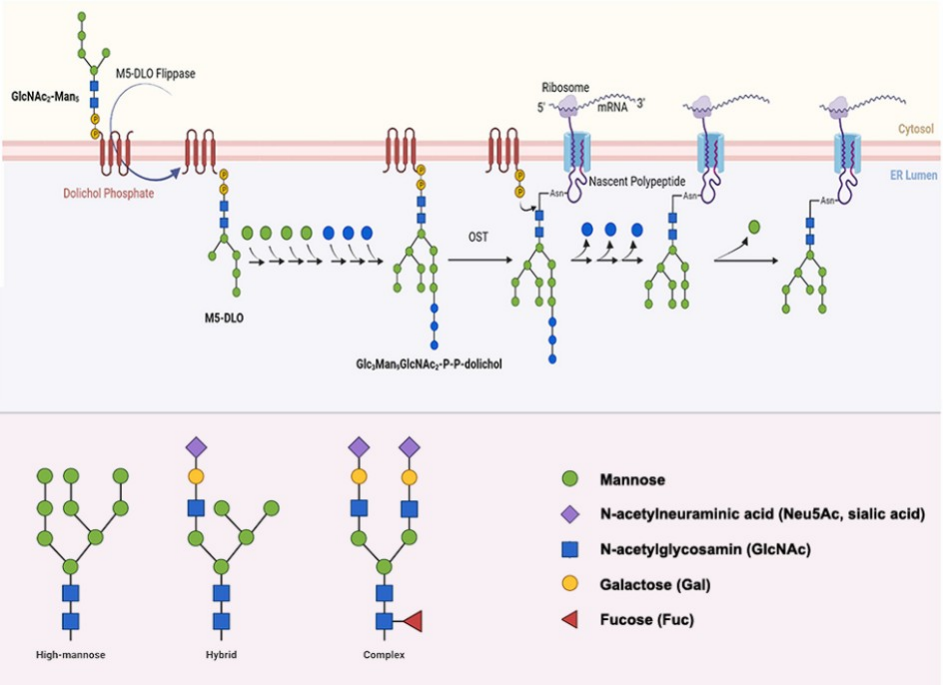
Figure 1 N-glycan biosynthetic pathway in the ER and the Golgi apparatus Reproduced from Ref. [14] with the permission of Elsevier.

N-glycosylation occurs within the endoplasmic reticulum (ER) and Golgi apparatus and is governed by a complex regulatory mechanism ( Figure 1) [13]. The process of N-glycosylation can be succinctly summarized as the formation of the lipid-linked oligosaccharide donor (LLO), the cotranslational transfer of the glycan to a nascent polypeptide, followed by processing within the ER and Golgi. In eukaryotes, LLO consists of a dolichol-pyrophosphate carrier (Dol-PP) linked to an oligosaccharide composed of fourteen glycan units. The biosynthesis of LLO starts with the transfer of N-acetylglucosamine-phosphate (GlcNAc-P) to dolichol-phosphate (Dol-P) at the cytoplasmic side of the ER membrane, which is catalyzed by ALG7. After several enzymatic reactions, the intermediate Dol-PP-GlcNAc2-Man5 was produced and then flipped into the ER lumen by an unknown mechanism, possibly but controversially involving the presumed flippase RFT1. In the lumen of the ER, Dol-P-P-GlcNAc2-Man5 is modified with mannoses via catalysis by the mannosyltransferases ALG3, ALG9, and ALG12 to form Dol-P-P-GlcNAc2-Man9. Dol-P-P-GlcNAc2-Man9 is then elongated by ALG6, ALG8, and ALG10 to give the fully assembled LLO Dol-PP-GlcNAc2-Man9-Glc3, the complete N-glycan precursor. The transfer of GlcNAc2-Man9-Glc3 from LLO to the Asn residue within the N-X-S/T (X cannot be proline) acceptor sequence of nascent proteins is catalyzed by oligosaccharyltransferase (OST), a multisubunit protein complex in the ER. The nascent carbohydrate-protein conjugate undergoes further processing in the ER, which usually involves the removal of glucose residues by glucosidase I and II and other quality control processes. Upon correct folding and attainment of native structures, the majority of proteins are exported from the ER to the Golgi apparatus in vesicles coated with cytosolic coatamer protein II (COPII) [14]. N-glycans on the side chains of proteins undergo further processing in the Golgi apparatus and are converted to the mature form. These processes include mannose trimming and various modifications catalyzed by mannosidases and several glycosyltransferases. GnT-I (MGAT1) and GnT-II (MGAT2) transfer GlcNAc to newly exposed Man. GnT-V (MGAT5) catalyzes the transfer of β1,6-linked GlcNAc to α1,6-Man, while GnT-III (MGTA3) transfers β1,4-linked GlcNAc to a β-linked core Man, thus producing a bisecting GlcNAc. The modification of the core fucose, also called α1,6-fucose, on GlcNAc is catalyzed by α1,6-fucosyltransferase (FUT8). In addition, GlcNAc residues can be further linked with β1,3- or β1,4-Gal and α2,3- or α2,6-Sia, which are catalyzed by β3GalTI or β4GalTI and ST3GalI or ST6GalI, respectively.

The biosynthesis of N-glycans is a complex process affected by both genetic and environmental factors [15]. In contrast to gene-encoded protein scaffolds, the glycans underlying glycoproteins lack a genomic template that defines their structures. Instead, the production of distinct glycoforms is regulated by a complex and dynamic network of glycan precursors, enzymes, and transporters [16]. Glycoenzymes, as proteins, essentially rely on transcriptional, translational and posttranslational mechanisms for the regulation of their expression and activity [16]. GWASs have revealed a number of genetic variants and mapped gene loci associated with the plasma or immunoglobulin G (IgG) N-glycome, highlighting the importance of genetic regulation in glycosylation processes [17]. Beyond genetic control, epigenetic changes influence the expression of a specific set of “glyco-genes”. Evidence from epigenome-wide association studies (EWAS) and several *in vitro* experimental studies suggested the impact of DNA methylation, histone modifications and microRNAs on glycosylation [ 18‒ 20]. Additionally, environmental factors can directly induce alterations in glycan structures. It has long been known that age is associated with changes in certain glycans [ 21, 22]. Other factors, such as body mass index (BMI), diet, smoking status, and alcohol consumption, have also been reported to affect N-glycosylation [ 22‒ 24].

### Biological significance of glycosylation

**Figure 2:**
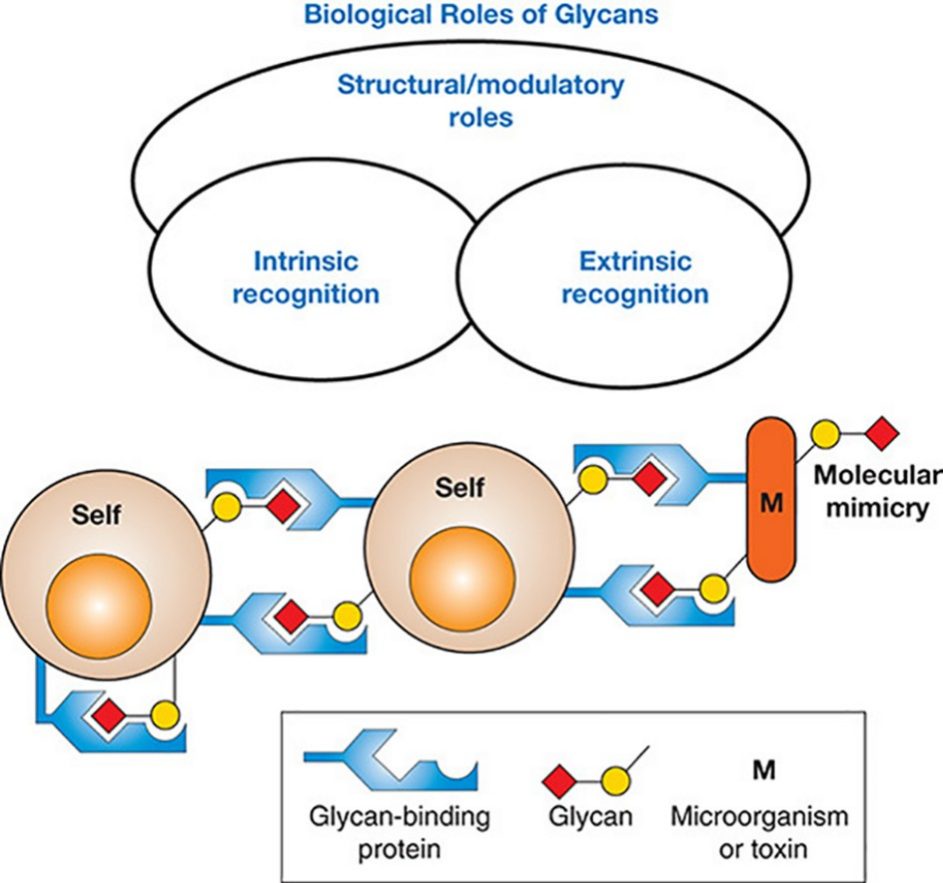
Figure 2 General classification of the biological functions of glycans Reproduced from Ref. [26] with permission from Cold Spring Harbor Laboratory Press.

N-glycans are pivotal in a myriad of biological processes, including protein folding, stability, and trafficking, as well as protein‒protein interactions, cell adhesion, signalling pathways, and immune responses. Organism-intrinsic and -extrinsic recognition by glycan-binding proteins underpins the extensive structural and regulatory roles of glycans ( Figure 2) [25]. In the context of protein glycosylation, the attachment of distinct glycans to the same glycosylation site, known as alternative glycosylation or microheterogeneity, significantly contributes to the structural diversity of these molecules and impacts their functionality.

Glycosylation of nascent proteins plays a crucial role in the regulation of protein folding and quality control throughout protein production. Proteins newly produced in the ER are transported to the Golgi apparatus only if they are correctly folded. Modification of nascent proteins with N-glycans not only enhances their solubility but also signals the recruitment of ER-resident lectin chaperones to regulate glycoprotein folding [26]. Misfolded glycoproteins undergo reglycosylation and re-bind to lectin chaperones to attain the correct folding state. In addition, N-glycosylation influences protein stability, either directly through property modifications or indirectly by stabilizing the secondary structure conformation of glycoproteins through the modulation of residue folding around the N-X-S/T sequence [ 27, 28]. Furthermore, various aspects of N-glycosylation are involved in the regulation of cell biology. Cell adhesion, one of the essential biological processes implicated in glycosylation, is dependent on the participation of adhesion receptors, including E-cadherin and integrin, cell-extracellular matrix (ECM) proteins such as laminin, and peripheral membrane proteins. GnT-III and GnT-V have been noted to have opposing effects on α5β1 integrin-mediated cell spreading and migration, indicating that the remodelling of glycosyltransferase-modified N-glycan structures can modulate cell adhesion and migration either positively or negatively. In addition, N-glycosylation plays a crucial role in receptor activation and signal transduction. For example, the epidermal growth factor receptor (EGFR), a tyrosine kinase glycoprotein, is glycosylated in its extracellular domain. N-glycosylation of EGFR has been demonstrated to mediate noncovalent interactions, thereby regulating receptor dimerization and binding with growth factors [29]. In addition, inhibiting N-glycosylation of receptor tyrosine kinases has been linked to defective activation and impaired signal transduction in cancer [30].

## Technologies for Glycomic Analysis

**Figure 3:**
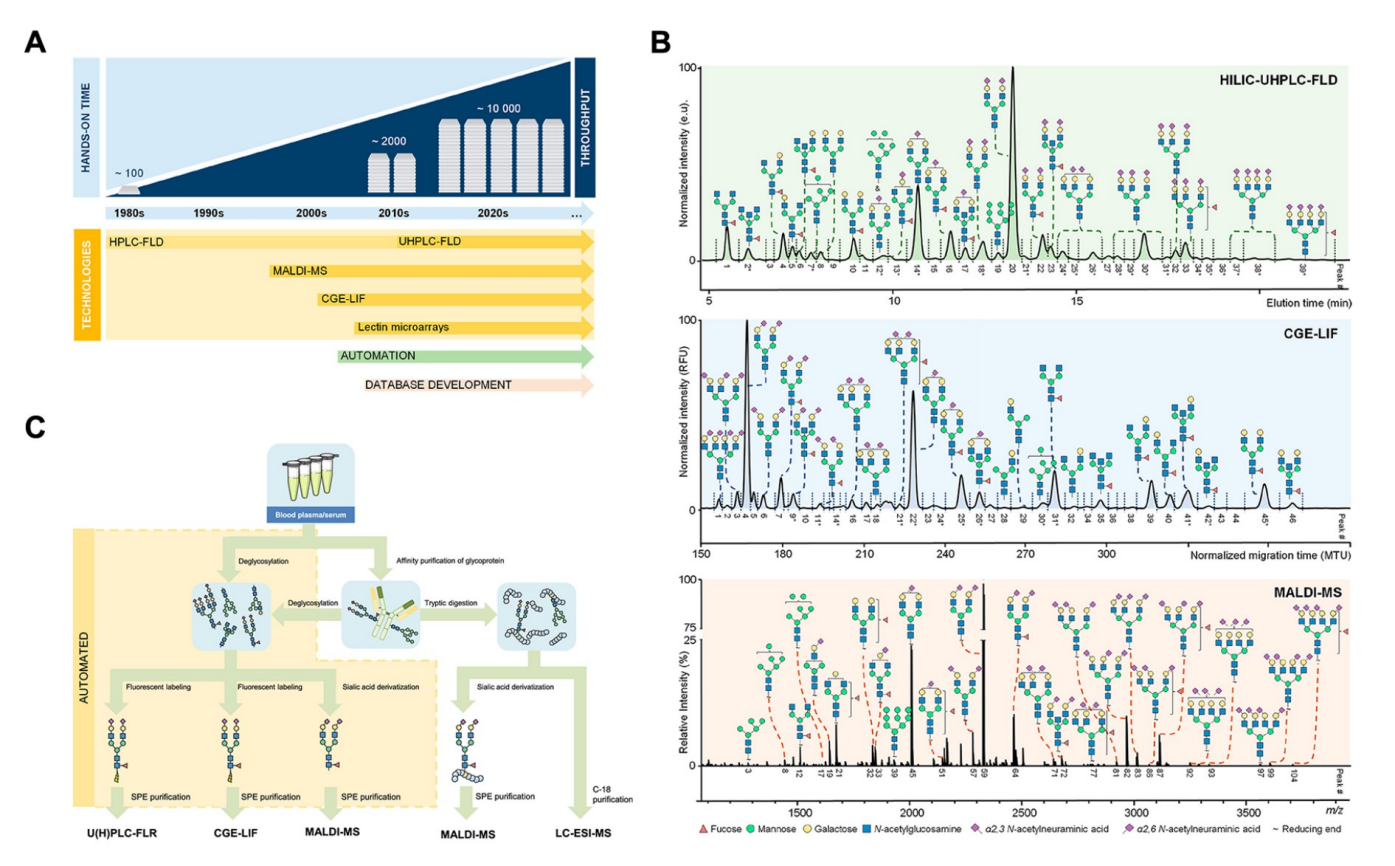
Figure 3 General technologies and workflow for glycomic analysis (A) Historical overview of high-throughput glycomic technologies applied for released N-glycan analysis. (B) Representative profiles of the total serum N-glycome using HILIC-UHPLC-FLD, CGE-LIF, and MALDI-MS. (C) General analytical workflow in glycomics. Reproduced from Ref. [32] with permission from the American Chemical Society.

Due to the extensive impacts of glycosylation on biological processes, glycomic analysis has been increasingly applied to investigate changes in glycomic profiles under different conditions, particularly changes in the levels of released glycans and intact glycopeptides. Current glycomic methodologies can be categorized into three main types: retention time-based glycan identification ( *e*. *g*., liquid chromatography), charge- or mass-based glycan identification ( *e*. *g*., capillary electrophoresis and mass spectrometry), and affinity-based identification ( *e*. *g*., lectin microarrays) ( Figure 3A,B) [31]. These methods offer distinct advantages in glycomic analysis and may be employed either individually or in combination to yield complementary information. The general procedures for glycomic analysis are similar, involving steps such as protein isolation, glycan or glycopeptide release and isolation, fluorescent labelling or chemical derivatization, purification, and ultimately detection and identification ( Figure 3C) [31]. Nevertheless, to obtain reliable and robust data, unique analytical conditions and data processing steps may be required for different technologies.

### Liquid chromatography

Liquid chromatography (LC), especially ultra-performance liquid chromatography (UPLC), has become a pivotal method for large-scale glycomic analysis. HILIC-UPLC-FLD, the most widely adopted LC-based approach, is the preferred method for routine analysis of protein glycosylation involving known glycan structures [32]. When combined with a fluorescence detector (FLD), UPLC enables the comprehensive characterization of complex glycan mixtures in a relatively short time. To characterize novel glycan structures, UPLC can be coupled with mass spectrometry (MS) to yield additional structural information.

The preparation of glycan samples for LC analysis typically involves enzymatic deglycosylation using PNGase F, fluorescent labelling of the released glycans, and subsequent purification steps. Regarding fluorescent labels, 2-AB remains the most popular choice because of its intense fluorescent signals. In recent years, many other fluorescent labels, including 6-aminoquinolyl-N-hydroxysuccinimidyl carbamate (AQC), procainamide, and RapiFluor-MS (RFMS), have been increasingly adopted [ 33‒ 35]. These labels have shown notable improvement in fluorescence intensity, sensitivity in fluorescence detection, and a substantial reduction in reaction time compared to 2-AB [35]. Glycan purification is typically carried out using hydrophilic interaction chromatography-solid phase extraction (HILIC-SPE) with various hydrophilic stationary phases, which effectively removes excess reagents and proteins from previous sample preparation steps [36]. Fluorescently labelled glycans are generally analyzed by HILIC-based LC techniques due to their remarkable ability to separate polar and hydrophilic analytes in an aqueous-organic mobile phase. After efficient separation based on glycan charges and sizes in the LC, glycans are quantified as relative percentage levels through distinct normalization [37]. For glycan identification, there are extensive HPLC/UPLC glycan databases containing the glucose unit (GU) value as an external standard [38]. The glycan structures can be assigned to each peak by matching the experimental values with the values in the databases. However, LC peaks and glycan structures often do not correlate on a one-to-one basis; in other words, a single peak may represent several possible glycans. In this case, exoglycosidase sequencing accompanied by MS has historically been used as a complementary approach to indicate specific structural features [39]. Additionally, HILIC coupled online to MS via electrospray ionization (ESI) enables the characterization of glycan structures in unknown samples [40].

### Capillary electrophoresis

Capillary electrophoresis (CE) has historically been employed primarily for DNA sequencing. However, since 2014, it has emerged as a powerful approach for the analysis of carbohydrates, particularly N-glycans [41]. Electroosmotic flow (EOF) and electrophoretic mobility constitute the two primary transport mechanisms in most CE separations, facilitating N-glycan separation based on their charge-to-size ratio [41]. In this regard, the majority of N-glycans can be effectively separated, except for those containing acidic sugars. Compared to UPLC and MS, CE not only offers greater sensitivity but also enables isomer separation and linkage analysis without compromising analytical robustness and cost. However, large CE-based glycan structural databases are lacking, which limits their application in large-scale glycomic analysis [41].

The sample preparation procedures for CE analysis resemble those for UPLC analyses. Through labelling with a charged fluorochrome, glycans can migrate in the electric field and be detected via laser-induced fluorescence (LID). Currently, 8-aminopyrene-1,3,6-trisulfonate (APTS) represents the preferred glycan label for CE analysis, surpassing other labelling options [ 42, 43]. Using APTS, glycans undergo covalent modification by a reductive amination reaction, resulting in a single-labelled product with nearly 100% labelling efficiency [44]. Subsequent purification of glycan samples is achieved through HILIC-SPE or, alternatively, through specific enrichment using magnetic beads [45]. In the field of glycan identification in CEs, several strategies have been devised, including the calibration of migration time using ladders and standards, exoglycosidase sequencing, and coupling with MS. Migration time alignment standards (coinjected bracketing standards) facilitate electropherogram alignment and GU unit assignment, enabling glycan identification and quantification while enhancing separation reproducibility [46]. Furthermore, the structures of separated glycans can be elucidated through comparisons of GU values in existing databases, utilization of exoglycosidase sequencing, or coupling with MS technologies [ 47, 48]. However, the integration of CE with MS remains challenging due to the incompatibility of the two technologies, which makes the structural annotation of glycan peaks rather complex [31].

### Mass spectrometry

MS is a powerful tool in glycomic analysis that significantly enhances the elucidation of glycan structures. Matrix-assisted laser desorption/ionization (MALDI) and ESI are preferred technologies because of their ability to preserve the structural integrity of analytes. Among these two MS strategies, ESI offers superior resolution and sensitivity compared to MALDI, rendering it more suitable for complex analyses when combined with precursor separation techniques. In contrast, MALDI-MS facilitates the rapid characterization of glycomic profiles in complex matrices with off-line glycan separation.

ESI-MS is usually referred to as a soft-ionization technique since it imparts relatively little energy to analyte molecules during desolvation. Glycan samples can be directly infused through either an off-line ESI source or by an on-line ESI source coupled to a separation medium such as LC or CE. In contrast to MALDI-MS, LC-ESI-MS can provide more information on glycosylation features, albeit with complex operations and lower throughput. The general workflow of LC-ESI-MS includes glycan release, purification, and labelling, followed by glycan separation and ESI-MS quantitation. For glycan separation, C18, HILIC, and PGC are the most frequently used techniques [ 40, 49, 50]. The individual procedures of sample preparation for LC‒ESI‒MS analysis largely depend on the separation technology. Dopant-enriched nitrogen gas (DEN gas) is usually utilized as an electrospray source to improve the desolvation and ionization efficiency of glycans and glycopeptides by enriching the nebulizing gas [51]. In addition, low-flow ESI conditions such as nano-LC-ESI-MS improve the sensitivity of glycopeptide and glycoprotein analysis, especially allowing the detection of low-abundance autoantibodies [ 52‒ 55].

MALDI-MS analysis combined with time-of-flight (TOF) or ultrahigh-resolution Fourier transform ion cyclotron resonance (FT-ICR) analysis has emerged as a critical tool for high-throughput analysis of glycans released from biofluids such as serum and plasma [ 56, 57]. Furthermore, MALDI imaging MS (MSI) allows for unbiased visualization of the spatial distribution of biomolecules in tissues, thereby facilitating the profiling of N-glycomes in complex samples [ 58, 59]. Sample preparation for MALDI analysis typically adheres to conventional glycomic procedures. Nevertheless, an essential additional step is the stabilization of negatively charged sialic acids to mitigate detection and quantification biases. Permethylation, along with other chemical derivatization methods, including methyl esterification, ethyl esterification, and methyl amidation, serves as an effective N-glycan labelling technique to counteract sialic acid instability during ionization [ 56, 60‒ 63]. Purification procedures for the labelled glycans before spotting on a MALDI plate are crucial for increasing the measurement sensitivity. The most commonly used methods for glycan purification are SPE based on HILIC and the use of hydrophobic porous graphitic carbon (PGC) [ 64, 65]. Alternatively, capturing bead affinity-based glycoblotting strategies are effective in enriching released glycans or glycoconjugates from complex samples, albeit with high reagent costs [66]. For detection, glycan samples are first mixed with an appropriate matrix and loaded onto a MALDI plate. After drying, the samples are irradiated by a pulsed laser to be ablated, desorbed, ionized, and finally accelerated into an MS analyser for analysis. The obtained MS data can be initially processed in the data acquisition software for smoothing, baseline correction, and recalibration. However, further offline data processing is essential for obtaining more reliable information. Several R packages, including MALDIquant and GeenaR, offer a comprehensive pipeline for the analysis of MALDI-MS data, which allows rapid spectral processing, quality control, feature extraction, and spectral clustering of MALDI-MS spectra [ 67, 68].

### Lectin microarrays

Lectin microarray technology was first described by Pilobello *et al*. in 2005 [69]. Lectins, which contain at least one noncatalytic site, allow for the specific and reversible binding of carbohydrates without altering their structure [70]. By immobilizing lectins with known glycan-binding specificity on a bioactivated solid surface in an array format, it is possible to detect the binding status of carbohydrate residues with corresponding lectins. Compared to other glycomic technologies, lectin microarrays offer advantages, including simple sample preparation and rapid high-throughput glycan profiling at subnanogram levels. However, the application of lectin microarrays is limited by the limited repertoire of naturally occurring lectins and unsatisfactory detection sensitivity. Furthermore, lectin microarrays are not able to provide quantitative analysis, necessitating complementary techniques such as MS for structure elucidation and glycan quantification [71].

By utilizing lectin microarrays, glycans in complex biological samples can be directly measured by specific lectins, obviating the need for protein digestion and glycan release procedures. The binding of carbohydrate residues to the corresponding lectins can be detected either directly, through prelabelling with fluorescent reagents, or indirectly, through glycoprotein targeting. Although the prelabelling of lectins may offer convenience, it can disrupt the interactions between glycoproteins and lectins, resulting in reduced sensitivity and the need for larger amounts of glycoproteins. In contrast, indirect detection through an antibody-overlay lectin microarray significantly enhances detection sensitivity, particularly for identifying weak conjugations. Following baseline correction and normalization of fluorescence intensities for each spot, the processed data can be subjected to analysis using suitable statistical methods.

### Ion mobility-mass spectrometry

In recent years, the integration of ion mobility spectrometry with MS (IM-MS) has emerged as a promising tool for glycan analysis [ 72, 73]. By measuring the time it takes for an ion to traverse a buffer gas under a low electric field, IM-MS offers insights into both the size and shape of ions [74]. As a result, isomers of multiple glycan classes and glycopeptides can be analysed without the need for derivatization or enzymatic reactions [ 75, 76].

Typically, IM-MS analysis consists of five steps, including sample introduction, compound ionization, IMS, MS, and ion detection. Vapour and liquid samples can be directly introduced or infused into the IM-MS, while solid samples require deposition on the sample plates before insertion. Various IM instruments are employed in glycomic analysis, including drift tube IMS (DTIMS), high-field asymmetric waveform IMS (FAIMS), travelling wave ion IMS (TWIMS), and trapped ion mobility spectrometry (TIMS) [ 77, 78]. These instruments differ in the nature of the electric fields applied to propel ions through the IMS cell during ion mobility separation. Specifically, a homogeneous electric field is utilized in DTIMS to directly determine the collision cross-sections of ions from measured drift times. In contrast, FAIMS applies alternating high and low electric fields, whereas TWIMS employs traversing potential pulses. The collision cross sections (CCSs) of ions reflect their characteristics and aid in identifying glycan structures and isomers. However, deriving meaningful and relevant structural candidates from calculated CCSs is challenging. Density functional theory (DFT)-based methods are ideal for determining meaningful theoretical gas-phase structures for oligosaccharides. However, the use of DFT entails high computational costs, especially for large molecules and glycans with numerous conformers [73]. Consequently, there is a pressing need for the development of new and enhanced tools for data analysis and the automation of glycan identification in future research.

### Glycan databases and data processing tools

With the development of glycomic technologies, a large amount of glycoscience data has been generated. However, the sparsity, heterogeneity, and field-specific encoding of glycomic data resulting from the high chemical diversity of glycans and the complexity of glycan analysis present great challenges in data processing and integration [ 79, 80]. Glycoinformatics, as an actively developing scientific branch, is called upon for assisting the scientific community in glycomic research. The Complex Carbohydrate Structure Database (CCSD, CarbBank, discontinued in 1996) was the first database in glycobiology for collecting glycan structures and original mass spectra. Currently available glycan structure-centered databases include the CFG glycan databases, KEGG glycan, GLYCOSCIENCE.de, CSDB (Carbohydrate Structure DataBase), UniCarbKB, JCGGDB (Japan Consortium for Glycobiology and Glycotechnology DataBase), GlycoStore, and the secondary database GlyTouCan, which has been summarized in previous reviews [79].

MS technology has been the most important technology for glycomic analysis. For the interpretation of MS output, recent advances in various searching software and tools have greatly accelerated high-throughput glycomic and glycoproteomic analysis. Among these, GlycoWorkbench represents the most widely used software to facilitate manual annotation of MS data by matching experimental MS/MS peak lists against theoretical fragments [81]. It not only allows automatic and fast interpretation of MS data but also provides a user-friendly interface for drawing various glycan structures. Recently, other software or online tools have been designed to complement existing tools. MSFragger-Glyco, a glycoproteomics mode of the MSFragger search engine, was used for fast and sensitive identification of N- and O-linked glycopeptides and open glycan searches [82]. Expanded searches through MSFragger-Glyco also revealed many sulfated and complex glycans that remained hidden in the original search. GlycReSoft is a software package for determining glycan profiles from LC/MS data. Supervised and unsupervised scoring methods can be implemented to enable the assignment of peaks to both known and unknown glycan compositions [83]. Another glycoproteomic tool, Glyco-Decipher, conducts glycan database-independent peptide matching and exploits the fragmentation pattern of shared peptide backbones in glycopeptides to improve spectrum interpretation and is able to identify more glycopeptide spectra and modified glycans than other tools [84]. PEAKS GlycanFinder is a database search and de novo sequencing tool [85]. Peptide-based and glycan-based search strategies are used to address the challenge of complex fragmentation of glycopeptides. In addition, a deep learning model in GlycanFinder aims to capture glycan tree structures and their fragment ions for de novo sequencing of glycans that do not exist in the database.

## Disease-relevant N-glycan Biomarkers

Glycans are potential biomarkers for the diagnosis and prognosis of multiple types of diseases. In this section, we summarize current findings in glycan biomarker discovery among diseases, including cancers, autoimmune diseases, nervous system disorders, and metabolic and cardiovascular diseases ( Table 1).

**Table:** **Table 1** Summary of potential glycan biomarkers for the diagnosis of diseases

Disease	Biomarker	Sample type	Method	Subjects ( *n)*	Performance	Reference
						
Colorectal cancer	CRCglycoA	Serum glycans	DSA-FACE	347	AUC: 0.92 Sensitivity: 25% Accuracy: 18% Better than CEA	[86]
	A 54-feature model containing glycans	Serum glycans	MALDI-MS	362	Accuracy: 87%	[87]
	The ratio of *m*/ *z* 1708/1914	Serum glycans	MALDI-TOF-MS	116	AUC: 0.899 Better than CEA and CA19-9	[88]
	A model containing glycans, sex and age	Serum IgG glycans	UPLC	1298	AUC: 0.755	[89]
	A panel comprising four GPs and CEA	Serum IgG glycans	UPLC	252	AUC: 0.844	[90]
	A panel of seven isomer-specific IgG N-glycans	Serum IgG glycans	PGC-nanoLC-ESI-MS/MS	119	AUC: 0.877 (independent validation set)	[91]
						
Breast cancer	A panel of five bi-fucosylated N-glycans	Serum glycans	LTQ-ESI-MS	134	AUC: 0.913 Better than CEA	[92]
	IDsys RT	Serum glycans	MALDI-TOF-MS	44	AUC: 0.91	[93]
	A model consisting of age, BMI, and two N-glycans	Serum glycans	MALDI-TOF-MS	298	AUC: 0.899 Sensitivity: 82% Specificity: 84%	[94]
	A panel of seven glycans	Serum IgG glycans	MALDI-MS	144	AUC:0.874	[95]
	*m*/ *z* 1591	Serum IgG glycans	MALDI-MS	119	AUC: 0.944	[96]
	*m*/ *z* 1794	Serum IgG glycans	MALDI-MS	119	AUC: 0.921	[96]
						
Lung cancer	All glycans and CRP	Serum glycans	HILIC WAX-HPLC	184	AUC: 0.942 Sensitivity: 87% Specificity: 88%	[97]
	A panel of eight glycans	Serum glycans	MALDI-TOF-MS	584	AUC: 0.86 (validation set)	[98]
						
Esophageal cancer	A panel of one IgG N-glycan and four derived features	Serum IgG glycans	UPLC	496	AUC: 0.807 (validation set)	[99]
						
Rheumatoid arthritis	FA1	Serum IgG glycans	HILIC-UPLC	323	AUC: 0.881	[100]
	Combination of three bisecting GlcNAc glycoforms	Serum IgG glycans	MALDI-TOF-MS	114	AUC: 0.81	[101]
						
Systemic lupus erythematosus	IGP48, age, sex and African admixture	Serum IgG glycans	UPLC	284	AUC: 0.842	[8]
						
Alzheimer’s disease	Combination of serum tau/bisecting GlcNAc ratio, APOE ε4 status, and Mini-Mental State Examination score	Serum glycans	enzyme-linked lectin assay	233	AUC: 0.81	[102]
						
Parkinson’s disease	A model featuring four glycan peaks	Serum IgG glycans	HILIC-UPLC	196	AUC: 0.97	[103]
						
Type 2 diabetes mellitus	Combination of nine altered N-glycans	Plasma glycans	UPLC	451	AUC: 0.805 Sensitivity: 79% Specificity: 73%	[104]
	Combination of 5 IgG glycans, 13 derived traits, sex, and age	Serum IgG glycans	UPLC	196	AUC: 0.734	[105]
	Combination of six IgG glycan traits and fasting blood glucose	Serum IgG glycans	UPLC	849	AUC: 0.859	[106]
						
Type 1 diabetes mellitus	Combination of 39 plasma glycans, sex, and age	Plasma glycans	HILIC-UPLC	432	AUC: 0.915	[107]
	Combination of 24 plasma IgG glycans, sex, and age	Plasma IgG glycans	HILIC-UPLC	432	AUC: 0.869	[108]

### N-glycan markers in cancer

Cancer represents a major public health challenge worldwide and remains one of the leading causes of death. Early detection and treatment of cancer are critical for improving patient prognosis and prolonging survival. Currently, biopsy, imaging, and tumor biomarker screening constitute the three most prominent tools for cancer detection. Tissue biopsy is widely regarded as the most reliable method for cancer diagnosis. However, limitations such as tissue inaccessibility and temporal/spatial heterogeneity present intractable challenges for biopsy analysis. Furthermore, imaging features frequently lag behind pathological status alterations. Consequently, there is an urgent need for innovative noninvasive testing methods to facilitate early cancer diagnosis and prognosis prediction.

Glycans are implicated in a wide array of fundamental biological processes associated with cancer, including inflammation, immune surveillance, cell adhesion, and cellular signalling and metabolism [11]. Changes in glycoproteins, glycosphingolipids, and glycans are recognized as hallmarks of human cancer. The most prevalent cancer-associated glycosylation alterations include enhanced sialylation, increased branched-glycan structures, and the overexpression of core fucosylation [11]. In fact, some serum glycoproteins/glycans have been clinically employed in diagnosing and monitoring various types of cancer. For instance, glycoproteins such as prostate-specific antigen (PSA), α-fetoprotein (AFP), cancer antigen 125 (CA125), CA15-3, and β-HCG (β-human chorionic gonadotropin) are employed as biomarkers for prostate, liver, ovarian, breast, and female reproductive system cancers, respectively [ 109‒ 113]. CA19-9, a versatile tumor biomarker in the digestive system, stands out as the sole fully glycol-composed marker and is also known as sialyl Lewis A glycotope (sLeA) [114]. However, the sensitivity and specificity of these cancer-associated glycoforms are relatively limited. On the one hand, the combination of existing biomarkers could enhance diagnostic efficacy [115]. On the other hand, the unsatisfactory specificity and sensitivity of current glycol biomarkers prompt the search for novel glycan biomarkers aimed at improving early and precancer diagnosis, as well as monitoring progression and recurrence. Here, we summarize the latest research advancements in N-glycan biomarkers in common cancers, underscoring the key findings and their potential clinical implications.

Colorectal cancer (CRC) is the third most frequently diagnosed cancer and the second leading cause of cancer mortality [86]. The tumorigenesis of CRC can extend over a prolonged period of 10‒15 years, commencing with aberrant glandular crypts and progressing gradually to precancerous lesions (polyps) prior to advancing to colorectal cancer [116]. Thus, the early and accurate identification of precancerous lesions in CRC is of significant clinical importance. Current approaches for early CRC screening primarily include colonoscopy, fecal tests, and blood-based screening. Carcinoembryonic antigen (CEA) and CA19-9 are the most widely used serum glycoprotein biomarkers for CRC, yet they lack sensitivity and specificity for early detection. To date, numerous studies have demonstrated aberrant glycosylation in CRC, providing new insights into the potential of glycan biomarkers for CRC.

Zhao *et al*. [87] reported a significant decrease in serum total core fucose residues and fucosyltransferase in patients with CRC, which was later corroborated by several studies [ 88, 117, 89]. Additionally, they developed two distinct panels of N-glycans to differentiate CRC from normal controls (CRCglycoA) and adenoma (CRCglycoB). Both models demonstrated superior diagnostic efficacy compared to CEA, with areas under the curves (AUCs) of 0.92 vs 0.81 for CRCglycoA and 0.81 vs 0.73 for CRCglycoB [87]. The ability of serum N-glycans to detect early CRC and/or advanced adenoma has received further support from additional studies. A machine learning model of N-glycans detected CRC and advanced adenomas with 87% accuracy and yielded a false prediction rate as low as 5/176 (2.8%) in an independent validation group [117]. The ratio of *m*/ *z* 1708/1914 demonstrated a greater AUC for CRC detection (0.889) than did conventional tumor markers, including CEA (AUC=0.766) and CA19-9 (AUC=0.615) [89]. Moreover, a higher *m*/ *z* 1708/1914 ratio was found to be correlated with advanced CRC stage and shorter survival, indicating the ability of N-glycans to predict CRC prognosis [89]. By coincidence, Wuhrer and colleagues observed that alterations in the serum N-glycome were associated with CRC survival [90]. Patients with an altered serum N-glycome exhibited a five-year survival rate of 46%, which was significantly lower than the 87% for those with an N-glycome similar to that of healthy controls [90]. Intriguingly, the changes in the serum N-glycome returned to a control-like profile after successful treatment, which suggested the potential of total serum N-glycans as a monitoring marker for CRC [90].

In addition to total serum N-glycans, glycan markers on specific glycoproteins have been investigated, with IgG being the most extensively studied. Lauc and colleagues reported that CRC was associated with a decrease in IgG galactosylation and sialylation and an increase in the core fucosylation of neutral glycans [91]. Additionally, Gu *et al*. [118] identified a potential early diagnostic biomarker comprising four glycosylation patterns and CEA for the simultaneous discrimination of advanced adenoma (AUC=0.847) and CRC (AUC=0.844) patients from healthy controls. Furthermore, isomer-specific and IgG subclass-specific N-glycosylation could play a critical role in revealing the premature pathology of CRC, thereby providing new perspectives for the screening of N-glycan biomarkers [ 119, 120]. Notably, the characterization of CEA site-specific glycosylation was expected to greatly improve the diagnostic and monitoring performance of CEA, given the substantial heterogeneity across individual N-glycosylation sites [ 121, 122].

Breast cancer (BC) is the most common malignant tumor worldwide [86]. Currently, mammography is the standard modality for breast cancer screening. However, there are challenges in effectively distinguishing between benign lesions and breast cancers, resulting in numerous unnecessary biopsies [123]. CA15-3 and CEA are the primary blood-based diagnostic and prognostic biomarkers for BC, albeit with limited sensitivity and specificity. Alterations in glycosylation play a key role in essential cellular behaviors during mammary development and BC progression [124]. In recent decades, abnormal glycoforms have been investigated for their potential to serve as dynamic indicators of systemic responses to BC [125].

Research has suggested that elevated levels of sialylated, branched, and fucosylated N-glycan structures are potential BC biomarkers for both diagnosis and progression [ 92, 93]. Several studies have compared the efficacy of N-glycan markers in BC diagnosis with that of established clinical markers. Ju *et al*. [95] created a panel of five bifucosylated N-glycans in human serum, achieving a cumulative AUC of 0.913, significantly surpassing that of CEA (AUC=0.794) [94]. Lee *et al*. [95] presented an effective MALDI-TOF platform named IDsys RT for breast cancer analysis, showing a maximum AUC of 0.91. In addition, an integrated analysis of serum N-glycans with age and BMI yielded a model consisting of age, BMI, and the intensities of two N-glycans, which clearly distinguished BC from benign lesions with an AUC of 0.899 and an optimal cut-off with 82% sensitivity and 84% specificity [96]. IgG N-glycans also demonstrated significant diagnostic capabilities in BC. A multiple logistic regression model based on Fc N-glycan was able to discriminate patients with BC from normal controls (AUC=0.874) [126]. In addition, two core-fucosylated and agalactosylated glycans were found to specifically distinguish stage II BC patients from normal controls, with AUCs of 0.944 and 0.921, respectively [127]. Notably, the application of N-glycan markers must consider the subtype of BC, given the varied N-glycan profiles exhibited across different subtypes [128]. Moreover, large-scale studies with independent validation sets are required to systematically assess the reliability and stability of these glycan biomarkers.

For monitoring the progression and recurrence of BC, N-glycan biomarkers have also shown great performance. Pierce *et al*. [129] discovered that elevated levels of agalactosylated biantennary glycans and sLeX-containing glycans were significantly correlated with lymph node metastasis, which is a critical determinant of survival in early-stage BC. In addition, high levels of glycans containing sLeX epitopes have been suggested to be associated with CTCs in peripheral blood [130]. Moreover, Matsumoto *et al*. [131] determined that plasma alpha-L fucosidase (FUCA) activity and an N-glycan at *m*/ *z* 2534 were predictive biomarkers for BC susceptibility to trastuzumab, underscoring the promise of N-glycan biomarkers in providing therapeutic guidance.

Lung cancer (LC) is the leading cause of cancer-related death worldwide [86]. The identification of biomarkers for early-stage LC detection and the discovery of appropriate therapeutic molecular targets are crucial for improving LC diagnosis and treatment. Early serum glycoproteomic analysis identified 107 glycopeptides in human serum and revealed that a subset of these glycopeptides differed significantly between LC patients and healthy individuals [97]. In a detailed glycomic analysis focusing on individual glycan structures, Ruhhak *et al*. [98] reported 29 differentially expressed glycan structures between LC tumor tissues and adjacent normal tissues. Specifically, an increase in oligomannose-type glycans and a decrease in fully galactosylated glycans were noted in the tumor tissues. Arnold and colleagues detected elevated levels of sLeX-containing, monoantennary, and highly sialylated glycans, along with decreased levels of core-fucosylated biantennary glycans, in the serum samples of 100 patients with LC in comparison to those of matching healthy controls [132]. Notably, the combination of the differentially expressed glyco-biomarkers demonstrated high sensitivity (85%) and specificity (86%) in predicting LC [132]. In our previous study, a panel of eight N-glycan structures showed satisfactory diagnostic performance for NSCLC, with an AUC of 0.86 in the validation set [133].

Esophageal cancer (EC) ranks as the sixth leading cause of cancer-related mortality. Esophageal squamous cell carcinoma (ESCC) is the predominant subtype of EC, yielding a 5-year survival rate of less than 30% [ 86, 134]. The poor prognosis of ESCC patients is largely attributed to late diagnosis caused by subtle symptoms in the early stages and insidious progression to the advanced stage [ 135, 136]. Therefore, identifying reliable early-stage biomarkers is crucial for improving the prognosis of patients with ESCC. Using proteomics and N-glycoproteomic site-mapping analysis, Gao *et al*. [99] identified a series of glycoproteins that are differentially expressed between ESCC tissues and adjacent normal tissues. These authors further reported that increased fucosylation of ITGB1 and CD276 contributed to the onset and progression of ESCC [99]. Moreover, Wu and colleagues found that IgG N-glycosylation profiles were independently correlated with esophageal precancerous lesions in squamous cell carcinoma, indicating the significant potential of N-glycans as early-stage biomarkers for ESCC [137]. In a follow-up study, researchers developed a predictive model incorporating one IgG N-glycan and four derived features, which achieved AUCs of 0.822 (95% CI: 0.786‒0.849) and 0.807 (95% CI: 0.758‒0.864) for ESCC diagnosis in the discovery and validation sets, respectively [138].

### N-glycan biomarkers in autoimmune diseases

Autoimmune diseases are a series of disorders characterized by an abnormal adaptive immune response against self-antigens, also known as “autoantigens” [139]. The etiology of autoimmune diseases has yet to be fully understood but is thought to involve the interplay between genetic susceptibility and environmental factors [ 139, 140]. Recognizing and differentiating autoimmune diseases can be challenging due to overlapping signs and symptoms between different diseases. Furthermore, laboratory examinations for autoimmune diseases are nonspecific and depend on a panel of inflammatory indicators, including the erythrocyte sedimentation rate (ESR), acute phase reactants, cytokines, and autoantibodies [141]. In recent decades, the emerging field of glycomics has demonstrated significant promise in advancing biomarker research across various autoimmune diseases. In particular, the Fc glycosylation pattern of IgG, a fundamental component of adaptive immunity, has considerable potential for identifying autoimmune diseases [142]. Here, we outline the current progress in the research on glycan biomarkers in autoimmune diseases.

Rheumatoid arthritis (RA), a chronic autoimmune disease, is characterized by synovial hyperplasia, autoantibody production, and cartilage and joint destruction [143]. Extra-articular manifestations also occur in RA, impacting other organs, such as the lungs, skin, and heart [143]. In 1985, Parekh *et al*. [144] demonstrated a differential serum IgG N-glycosylation pattern between patients with RA and normal individuals, which represented the first study in this field. They identified significantly decreased galactosylation in RA patients by evaluating the primary sequences of approximately 1400 oligosaccharides from 46 IgG samples. With the rapid development of novel characterization techniques in glycomics in the past decade, the reduction in IgG galactosylation levels in RA has been verified in dozens of studies, making it the first well-established glycan feature of RA [ 145, 146]. In addition to reduced IgG galactosylation, other glycomic alterations, including decreased sialylation and increased fucosylation, were also observed in RA patients, as reviewed by Flevaris and Kontoravdi [100]. Moreover, it has been suggested that IgG galactosylation and sialylation levels are negatively correlated with RA activity [147]. Intriguingly, after treatment with effective therapeutic drugs, an increase in galactosylated and sialylated IgG glycans was observed with RA improvement [147]. Several studies have estimated the performance of IgG glycans as biomarkers for RA. Sebastian *et al*. [101] identified an IgG N-glycan structure, FA1, as a sensitive biomarker for distinguishing RA patients from matched controls in the Chinese Han population, with an AUC of 0.881. Through the development of a microfluidic chip-based method, Wang and colleagues identified two high-potential IgG sulfated N-glycan biomarkers for the classification of both rheumatoid factor (RF)-positive and -negative RA patients, as well as anti-citrullinated protein antibody (ACPA)-positive and -negative RA patients [148]. Additionally, Sun *et al*. [149] combined three bisecting GlcNAc glycoforms, yielding great performance for distinguishing between RA and osteoarthritis (AUC=0.81).

Systemic lupus erythematosus (SLE) is a chronic, multiorgan autoimmune disorder that predominantly affects women and certain ethnic groups [150]. Lupus nephritis (LN) is one of the most common and severe clinical manifestations of SLE, affecting up to 60% of SLE patients and remaining a major risk factor for overall mortality in SLE patients [151]. Similar to findings in RA, a reduction in IgG galactosylation has also been widely reported in patients with SLE. This commonality suggested that IgG agalactosylation may represent a broad phenomenon linked to the compromised immunosuppressive capability of circulating IgG rather than a disease-specific hallmark [152]. In addition, Vučković *et al*. [153] reported decreased IgG galactosylation, sialylation, and core fucosylation, as well as increased bisecting GlcNAc, in patients with SLE compared to healthy controls. Moreover, these alterations were associated with the severity of symptoms, highlighting the significance of the IgG N-glycome for the monitoring of SLE treatment [153]. IKZF1, which has been strongly linked to SLE development, was identified as a gene locus associated with the IgG N-glycome in a meta-analysis of GWAS findings [ 8, 122]. Hence, Lauc *et al*. [8] further explored the potential of IKZF1-associated N-glycans as biomarkers for SLE and demonstrated the substantial power of fucosylated bisecting N-glycans to differentiate between healthy controls and SLE patients (AUC=0.842).

In recent years, there has been a growing interest in studying glycan biomarkers for identifying LN in SLE patients. Alves *et al*. [154] initially revealed aberrant mannosylation patterns in LNs by comprehensive glycomic analysis of kidney tissues. Subsequently, Wolf *et al*. [155] discovered 72 altered urine N-glycans in LN patients compared to healthy controls (HCs), with three N-glycans showing significant sex-based differences. This finding indicated that the urine N-glycome may act as a definitive biomarker for LN, potentially reflecting renal disease activity. A plasma IgG N-glycome study involving SLE patients with or without LN revealed significantly lower levels of sialylated, galactosylated, and fucosylated glycans and higher levels of bisected GlcNAc in patients with LN [156]. Furthermore, a combination of three glycans with anaemia indicators proved effective in identifying the presence of LN, achieving an AUC of 0.792 (95% CI: 0.727‒0.858) [156].

IgA nephropathy (IgAN), or Berger disease, is the most prevalent primary glomerular disease worldwide [157]. IgAN pathogenesis is hypothesized to be initiated by elevated levels of circulating galactose-deficient IgA1 (gd-IgA) [158]. Subsequently, gd-IgA is recognized by antiglycan autoantibodies, which leads to the formation of immune complexes capable of depositing in the kidney, causing glomerular inflammation, complement activation, and kidney injury [158]. Therefore, N-glycosylation plays a crucial role in the pathogenesis of IgAN. Dotz *et al*. [159] reported various N-glycosylation structural differences in IgA1 and IgA2 between patients with IgAN and patients with normal glomerular function, including differences in galactosylation, sialylation, bisection, fucosylation, and N-glycan complexity. With regard to disease prediction, glycopeptides and derived traits were found to serve as better predictors of IgAN than gd-IgA levels, underscoring the potential utility of glycoproteomics in clinical settings [159].

### N-glycan markers in nervous system disorders

Alzheimer’s disease (AD) is a chronic neurodegenerative condition resulting from neuronal damage in the brain, which is believed to begin at least 20 years prior to the emergence of symptoms and recognizable dementia [160]. Currently, treatments for AD can only decelerate this advance without reversing it, highlighting the importance of early diagnosis and intervention in refining the quality of life of patients with AD. Clinical diagnostic methods for AD predominantly depend on measuring amyloid beta (Aβ) levels in cerebrospinal fluid (CSF) or plasma and brain imaging via positron emission tomography (PET) [ 161, 162]. However, these techniques are limited by their invasiveness, high cost, and unsatisfactory sensitivity. Therefore, novel biomarkers with better diagnostic performance are urgently needed to achieve early diagnosis of AD.

Dysregulated glycosylation in AD brains has been shown to impact a variety of biological processes, including neuroinflammation, cell adhesion, and cell signaling [ 163, 164]. Furthermore, numerous studies have identified aberrant glycosylation patterns in AD-associated proteins such as amyloid precursor protein (APP), tau, beta-secretase 1 (BACE1), and nicastrin (NCSTN), highlighting the potential for N-glycan biomarkers in early AD diagnosis [ 108, 165]. Palmigiano *et al*. [165] noted an elevated level of bisected N-glycans in the CSF of patients with both AD and mild cognitive impairment (MCI), suggesting that changes in CSF glycosylation may precede the clinical onset of AD. Schedin-Weiss and colleagues subsequently determined that alterations in CSF N-glycans correlated with the levels of phosphorylated tau and total tau in CSF, reinforcing the association between N-glycosylation, neurodegeneration, and tau pathology in AD [166]. Nevertheless, due to the trauma involved in lumbar punctures, CSF samples are not preferred for clinical testing compared to blood samples. Gizaw *et al*. [102] compared the N-glycome across brain tissue, serum, and CSF samples between AD patients and healthy controls. They noted significant increases in serum and CSF bisect-type N-glycans and multiply branched glycoforms in the AD group, whereas N-glycan profiles in brain tissues showed little variation between AD patients and healthy controls [102]. Consistent findings were also reported in a later study in which serum N-glycans, rather than cortical N-glycans, had stronger associations with AD [102]. To determine whether serum-based glycomic profiling may be a useful approach for biomarker discovery in AD and related dementias, Tena et al. performed a serum-based glycopeptide analysis and identified an increased abundance of serum nonfucosylated IgG1 and IgG2 in AD [167]. Further PLS-DA analysis of serum glycopeptides successfully separated AD patients from the control group [167]. Another longitudinal study emphasized the diagnostic value of the baseline serum tau/bisecting GlcNAc ratio in predicting future AD (AUC=0.68), with a mean time to AD diagnosis of 9.1 years [103]. Remarkably, the AUC was significantly improved to 0.81 when APOE ε4 allele status was combined with the baseline Mini-Mental State Examination (MMSE) score [103]. A recent study suggested a major difference in the Fc N-glycosylation pattern of serum natural autoantibodies (nAbs) against the pathologic isoform of Aβ (Aβ42) between AD and control groups, yet its diagnostic value remains to be validated [168].

Parkinson’s disease (PD) is recognized as the second most prevalent neurodegenerative disorder following AD and manifests with classic motor symptoms such as resting tremor, rigidity, bradykinesia, and postural instability [134]. To date, accurate diagnostic markers for PD are lacking, and PD identification is primarily reliant on clinical symptoms and signs. In a case-control study, Russell *et al*. [169] examined plasma IgG glycans in PD patients and explored the diagnostic potential of N-glycan markers for PD. Significant differences in glycan peaks were identified between PD patients and healthy controls. Moreover, a logistic regression model featuring four glycan peaks achieved satisfactory performance in distinguishing PD patients from controls, with an AUC of 0.97 (95% CI: 0.95–0.99) [169]. In addition, Xu *et al*. [170] observed elevated levels of glycans with core fucose, sialic acids, and bisecting GlcNAc in patients with PD. Intriguingly, in a follow-up study on urine samples, a decrease in the abundance of nearly all identified urine N-glycans, including those increased in serum samples, was noted in PD patients [171]. This finding suggested that alterations in the N-glycome may be distinct across different biological samples. In addition, specific changes in N-glycosylation of PD-associated glycoproteins, including ceruloplasmin and clusterin in serum, as well as 1-microglobulin/bikunin and uromodulin in urine, were identified [171]. In general, N-glycosylation plays a role in PD pathogenesis and is a potential disease-specific biomarker for PD.

### N-glycan markers in metabolic and cardiovascular diseases

Diabetes mellitus (DM) is a chronic condition characterized by hyperglycemia, with an accompanying risk of long-term complications impacting the kidneys, eyes, heart and nervous system [172]. DM is broadly classified into type 1 DM (T1DM), T2DM, and gestational diabetes mellitus [172]. T1DM is caused mainly by the destruction of pancreatic islet function and insufficient insulin production, while T2DM is characterized by insulin resistance and is the predominant type of DM [172]. The clinical diagnosis of diabetes relies on measurements of plasma glucose concentration and HbA1c levels. However, accurately identifying the specific type of diabetes in individuals continues to be challenging [173].

Using a chromatographic approach, Keser *et al*. [104] observed increased branching, galactosylation, and sialylation of plasma protein N-glycans in individuals at greater risk of developing T2DM. This finding suggested a correlation between increased plasma N-glycome complexity and increased T2DM risk, which was subsequently confirmed in several studies [ 174, 105, 106]. Remarkably, Adua et al. combined nine altered N-glycans to predict T2DM status among Ghanaians, resulting in an AUC of 80.5%, with a sensitivity of 79% and a specificity of 73% [105]. In addition, IgG N-glycans also exhibited robust predictive power as biomarkers for T2DM, and the AUCs were further improved when combined with either fasting blood glucose (FBG) or age and sex [ 175, 107]. Moreover, a recent Mendelian randomization study provided evidence at the genetic level that IgG N-glycans are causally associated with T2DM, which supported the biological foundation of IgG N-glycan biomarkers in predicting T2DM [176].

In T1DM, Rudman *et al*. [7] reported an increase in plasma and IgG high-mannose and bisecting GlcNAc structures, along with a decrease in monogalactosylation, which was distinct from the N-glycan alterations identified in T2DM. Moreover, diagnostic models including N-glycans, age, and sex yielded notable discriminative power between children with T1DM and their healthy siblings, with AUCs of 0.915 and 0.869 for plasma and IgG N-glycans, respectively [177]. Notably, individual differences in plasma N-glycosylation have been more extensively studied in the context of T1DM complications. A significant increase in alpha3-fucosylation of the acute phase protein alpha1-acid glycoprotein (AGP) was observed in T1DM patients with increased urinary albumin excretion. This suggested that glycosylation could serve as an additional marker for the development of vascular complications in patients with TIDM [178]. In addition, N-glycan alterations were also observed in patients with other T1DM complications, including renal impairment, severe albuminuria, hypertension, and T1DM retinopathy [ 179, 180].

Cardiovascular diseases (CVDs), the predominant noninfectious diseases globally, involve a spectrum of conditions affecting the heart and blood vessels [181]. It has been demonstrated that IgG glycans may play a role in the atherosclerosis associated with CVD. Specifically, elevated levels of IgG bisecting GlcNAc were positively linked to the presence of atherosclerotic plaques in carotid and femoral arteries, whereas the lack of bisecting GlcNAc and increased sialylation served as protective factors [182]. Moreover, Birukov *et al*. [106] identified sex-dependent associations between IgG glycosylation patterns and CVD risk.

Coronary artery disease (CAD) ranks as the most common form of CVD. A recent study comparing 316 CAD- and 156 CAD+ patients revealed differences in IgG N-glycan composition, showing that sialylated N-glycan structures negatively correlate with CAD [183]. In addition, IgG N-glycans have shown potential as biomarkers for atrial fibrillation (AF), a condition resulting from both abnormal electrical signalling in the heart and a susceptible heart substrate [184]. Specifically, AF patients were found to have higher levels of plasma and IgG oligomannose and lower levels of IgG bisecting GlcNAc than controls.

## Conclusion and Perspectives

Glycans are integral to a variety of physiological and pathological cellular functions, including cellular interactions, signal transduction, and immune responses. Advanced glycomic analysis has revealed alterations in glycan profiles, particularly the N-glycome, across a wide spectrum of common diseases. Compared with traditional biomarkers, various glycans have shown superior sensitivity and specificity in the diagnosis and monitoring of diseases. However, only a limited number of glycan biomarkers have been approved for clinical use. Several challenges remain to be addressed. To confirm the reliability and stability of potential glycan biomarkers for disease detection, longitudinal studies involving large populations with independent validation sets are essential. In addition, glycan alterations are sometimes similar across different diseases, which presents an obstacle to their application as specific biomarkers under certain conditions. In this context, data-driven predictive modelling and artificial intelligence (AI)-based glycomic analysis could contribute to finding reliable and specific N-glycan biomarkers and facilitating clinical translation [100]. Furthermore, effective and high-throughput sampling procedures with high repeatability are crucial for clinical application. In addition, it might be challenging for clinical chemists with limited experience in glycomics to interpret glycomic data. Thus, concise and user-friendly software for data processing and analysis is needed. In summary, glycosylation has deepened the understanding of disease pathology and promoted the development of more precise diagnostics and treatments. Ongoing research continues to uncover the considerable potential of glycan biomarkers in diseases and address issues in clinical translation, promising for markedly enhancing the diagnosis and monitoring of complex diseases in the short term.
